# Association between the Catechol O-Methyltransferase (COMT) Val158met Polymorphism and Different Dimensions of Impulsivity

**DOI:** 10.1371/journal.pone.0073509

**Published:** 2013-09-10

**Authors:** Leandro Fernandes Malloy-Diniz, Guilherme Menezes Lage, Simone Becho Campos, Jonas Jardim de Paula, Danielle de Souza Costa, Marco Aurélio Romano-Silva, Débora Marques de Miranda, Humberto Correa

**Affiliations:** 1 Laboratório de Investigações Neuropsicológicas (LIN), Universidade Federal de Minas Gerais, Belo Horizonte, Minas Gerais, Brazil; 2 INCT de Medicina Molecular, Faculdade de Medicina, Universidade Federal de Minas Gerais, Belo Horizonte, Minas Gerais, Brazil; 3 Departamento de Saúde Mental, Faculdade de Medicina, Universidade Federal de Minas Gerais, Belo Horizonte, Minas Gerais, Brazil; 4 Departamento de Pediatria, Faculdade de Medicina, Universidade Federal de Minas Gerais, Belo Horizonte, Brazil; Universidad de Granada, Spain

## Abstract

**Background:**

Impulsivity is a multidimensional construct which has been associated with dopaminergic neurotransmission. Nonetheless, until this moment, few studies addressed the relationship between different types of impulsivity and the single nucleotide polymorphism caused by a substitution of valine (val) with methionine (met) in the 158 codon of the Catechol-o-Methyltransferase gene (COMT-val158met). The present study aimed to investigate the association between val158met COMT polymorphism and impulsive behavior measured by two neuropsychological tests.

**Methodology/Principal Findings:**

We administered two neuropsychological tests, a Continuous Performance Task and the Iowa Gambling Task were applied to 195 healthy participants to characterize their levels of motor, attentional and non-planning impulsivity. Then, subjects were grouped by genotype, and their scores on impulsivity measures were compared. There were no significant differences between group scores on attentional and motor impulsivity. Those participants who were homozygous for the met allele performed worse in the Iowa Gambling Task than val/val and val/met subjects.

**Conclusions/Significance:**

Our results suggest that met allele of val158met COMT polymorphism is associated with poor performance in decision-making/cognitive impulsivity task. The results reinforce the hypothesis that val and met alleles of the val158met polymorphism show functional dissociation and are related to different prefrontal processes.

## Introduction

Impulsivity has been considered both a personality trait and a pattern of response with different presentations and outcomes. Moeller and colleagues [1], for instance, argue that impulsivity encompasses swift action without forethought or conscious judgment, behaviour without sufficient thought, and the tendency to achieve a response without planning. Considering this broad meaning, an increasing number of studies have investigated impulsivity as a multidimensional construct. Even in the absence of a clear and consistent definition of impulsivity, some authors have proposed that different types of impulsive behaviour might be independent, both in terms of phenomenological and neurobiological characteristics [2–4].

Bechara and Van der Lynden [3] have proposed that impulsive behaviour is composed of at least two main dimensions related to different neurobiological systems. The first dimension is the impaired capacity to inhibit behaviour, called motor impulsivity. The second dimension is the inability to delay gratification and the orientation towards an immediate reward rather than a more advantageous but delayed reward. This dimension is usually called impulsive decision-making or cognitive impulsivity [5,6]. According to Bechara and Van der Lynden [3], the two dimensions are controlled by different neural substrates. Posterior regions of the orbitofrontal/ventromedial prefrontal cortex, including the basal forebrain, are associated with motor impulsivity, while the anterior part of the orbitofrontal/ventromedial prefrontal cortex, including the frontal pole, is associated with cognitive/decision-making impulsivity. Another cognitive expression of impulsivity [2,5] relates to working memory and the inability to inhibit irrelevant information held in working memory to focus on the task at hand, which is linked to the dorsolateral prefrontal cortex.

A relationship among dimensions of impulsive behaviour has also been found at the molecular level. Components of the monoaminergic and other neurotransmitter systems seem to play a differential role in impulsivity expression [4]. The dopaminergic system seems to play a pivotal role in control of impulsive behaviour [7,8]. There is evidence that low dopaminergic activity affects different types of impulsivity, such as those related to inhibitory control [9] and decision-making [10].

A key gene polymorphism that may influence both the dopaminergic system and impulsivity is the catechol-O-methyltransferase enzyme (COMT) [11]. It accounts for more than 60% of the metabolic degradation of dopamine in the frontal cortex [12]. A functional polymorphism that modulates COMT activity is *rs4680* (val158met), which produces a substitution of the amino acid methionine (met) for valine (val) at codon 158 in the COMT gene. The met allele enzyme has one-third to one-half of the activity of the val allele variant [13]. Therefore, individuals homozygous for the met allele are expected to have increased levels of endogenous dopamine in comparison to individuals with two val alleles. The role of COMT in the dopaminergic system is of particular importance in the prefrontal cortex because the dopamine transporter is uncommon in this cortical region [14].

Several studies aiming to assess functional differences between subjects with different val158met polymorphisms have shown that those homozygous for Met alleles show better performance in cognitive functions related to frontostriatal networks, such as executive function [15]. The results are nonetheless controversial, and there are some negative results concerning the association between the met allele and executive functions [16,17].

Concerning impulsivity, Forbes and colleagues [18] did not find a relationship between attentional, motor and non-planning dimensions of impulsivity measured by the Barratt Impulsiveness Scale-11 and the COMT allele in a healthy adult sample. The same result was reported by other researchers in a sample of adolescents [19]. It is interesting to note that both studies used self-reported measures of impulsive behaviour. Another possible way to investigate the hypothetical association between COMT alleles and impulsivity is using direct behavioural tests^11^. These are more reliable than self-report questionnaires because behavioural tests are independent of recall and interpretation of past behaviour [20]. In previous studies, we have proposed a multidimensional assessment of impulsivity based on two computerised tasks: the continuous performance test (CPT) and the Iowa Gambling Task (IGT) [6,20–23].

Despite previous evidence that higher COMT metabolism was associated with better performance in CPT [24], its is still controversial the relationship between COMT alleles and performance in IGT task. Van de boer et al. [25], reported an association between val allele and better performance of healthy females in IGT. Similar results were founded by Roussos et al. [26] in a study of the relationship between planning and emotional decision making and the rs4818 polymorphism. The authors found that those subjects who carry two copies of the allele related to lower prefrontal dopamine levels present better performance in the decision-making task. Nonetheless, some studies did not find a relationship between COMT val158met polymorphism and IGT performance [27,28].

The aim of this study was to investigate the hypothesis that the functional differences between the met and val COMT alleles affect impulsive behaviour. In particular, we aimed to assess the hypotheses that homozygosity at the met allele leads to better performance in at least two tasks related to frontostriatal circuits in a healthy subject’s sample.

## Methods and Procedures

### Participants

The study was approved by the Universidade Federal de Minas Gerais ethics committee and was conforming to the principles expressed in the Heksinki Declaration. All subjects signed a study informed consent. We assessed 195 healthy adults from the city of Belo Horizonte, Brazil. The participants were invited by local announcements at two Universities and the researcher’s network. The inclusion criteria were age between 18 and 60 years, at least 11 years of formal education, familiarity with the computer/mouse interface, no severe sensory or motor impairments and no history of psychiatric or neurological disorders. To ensure lack of psychiatric conditions, the MINI neuropsychiatric interview [29] was used to screen for DSM-IV axis I disorders. Only subjects without psychiatric disorders were included in the sample. From 195 participants enrolled in the study, three were excluded due to invalid test data, reducing the sample size to 192. This sample size would allow the detection of a large effect with 99%, a moderate effect with 95% and a small effect with 65% accuracy [30]. The mean age of the participants was 28.12 (SD =10.15), ranging from 18 to 60 years, and the mean length of formal education was 11.89 (SD =1.72), ranging from 11 to 17 years. There were more female (n=109) than male (n=83) participants.

### Impulsivity Assessment

The neuropsychological assessment has been described elsewhere (6,20-22]. The tasks used were the CPT [31], which provides measures of sustained attention and impulsiveness, and a computerised Brazilian version of IGT [6,21].

In the CPT, subjects have to press the spacebar when any letter (except the letter X) appears on the screen. Omission errors occur when the individual does not press the spacebar when a letter (except X) appears on screen, and reflect instances when the participant is not attentive to the target stimuli. A commission error occurs when the subject presses the spacebar when the letter X appears on screen, reflecting a flaw in motor response inhibition. These two measures are similar to attentional and motor impulsiveness, respectively, in Barratt’s model of impulsiveness [6]. Thus, we used commission and omission errors as dependent measures to evaluate motor and attentional impulsivity.

In the IGT, subjects have to choose one card at a time from four available decks (A, B, C and D). The task requires the subjects to make 100 choices (100 trials), and in each trial, subjects may win or lose a certain amount of money. During the instructions, subjects are told that some decks are more advantageous than others, but they do not know which are the better decks. After each choice, subjects receive feedback on the computer screen telling them how much money they won or lost. Using the feedback, the subject has to avoid decks that yield high immediate gains but lead to large future losses (decks A and B) and choose the decks that lead to small immediate gains but avoid substantial losses throughout the task (decks C and D). One hundred choices are divided into five blocks, with twenty choices each. This type of register is important to verify changes in the pattern of choices during the task, such as the learning curve. A total Netscore (number of cards selected from the advantageous “good” decks minus the disadvantageous “bad” decks) was used. This test is a good model for studying non-planning/cognitive impulsivity [6].

### COMT genotyping

The polymorphism was assessed by a standard procedure previously reported [32]. Briefly, genomic DNA was extracted from blood samples using the high salt method [33]. The COMT functional polymorphism (val158met, *rs4680*) were purchased in a made-to-order from Applied Biosystems®. Genotyping was performed using a real-time PCR system in the allelic discrimination mode (Stratagene Mx3005 – MxPro QPCR- Software, 2007) using the TaqMan Genotyping Master Mix (Applied Biosystems, Foster City, CA). PCR parameters included an initial denaturation at 95°C for 10 min, followed by 50 cycles at 95°C for 15 seconds and 60°C for 1 minute. Each reaction contained 3.5 µl of Mix, 0.1 µl of probe, 3.4 µl of deionised water and 1.0 µl of DNA. Researchers involved in genotyping were blind to neuropsychological results, and researchers involved in neuropsychological assessments were blind to the genotyping results. COMT genotype was coded as a categorical variable (met/met, met/val and val/val) for further analysis.

### Statistical Procedures

The distribution of impulsivity data, assessed by the Kolmogorov-Smirnov Test, violated the normal distribution for the CPT variables (p<0.05) but not for the IGT net score (p=0.667). Data transformations were performed aiming to normalise CPT scores, but none succeeded, so non-parametric tests were used for the CPT analysis and parametric tests for the IGT analysis. First, the influence of age, education and gender on test performance was assessed by correlation analysis. Variables significantly related to neuropsychological performance were controlled for the following procedure. To assess the influence of COMT genotype on impulsivity, Generalized Linear Models were adopted for the CPT data. The models for ordinal response (logistic) were built independently, with the CPT measures as dependent variables, the COMT polymorphism as a factor and, if necessary, sociodemographic variables as covariates. Main effects and interactions between factors were computed. The procedure for IGT data was similar, but a parametric procedure was performed. Statistical significance was established at 0.05. All statistical procedures were performed using SPSS 19.0 [34].

## Results

The COMT genotype distribution was under Hardy-Weinberg equilibrium (χ^2^=2.49, p=0.114). Participant descriptions and neuropsychological test data stratified by COMT Genotype are shown in Table 1. No differences between age, education and sex were found between the three groups (p>0.05).

**Table 1:** Participants description and performance on neuropsychological impulsivity measures.

	**met/met (n= 39, F=22)³**			**met/val (n=84, F=45)³**			**val/val (n=69, F=41)³**		
	**Mean (SD)**	**Median**	**Min-Max**	**Mean (SD)**	**Median**	**Min-Max**	**Mean (SD)**	**Median**	**Min-Max**
**Age¹**	28.33 (10.91)	24	18-60	28.01 (9.93)	24	18-60	28.14 (10.13)	24	18-75
**Education²**	11.56 (1.52)	11	11-16	11.85 (1.54)	11	11-17	12.13 (2.01)	11	11-17
**CPT Omissions**	3.77 (5.29)	2	0-26	3.86 (5.85)	2	0-30	2.35 (3.85)	1	0-26
**CPT Comissions**	13.72 (7.05)	14	2-28	12.67 (7.40)	12	0-29	12.57 (7.78)	10	2-33
**CPT Response Time**	366.44 (56.75)	354	293-517	378.88 (68.07)	371	273-604	377.80 (74.10)	373	265-698
**IGT - Netscore**	7.46 (19.46)	8	-26-58	9.52 (20.57)	6	-38-60	19.48 (25.48)	20	-62-64

Group differences: 1 - Age (F=0.13, p=0.987); 2 – Education (F=1.40, p=0.248), 3 – Sex (χ^2^=0.52, p=0.768)

CPT: Continuous Performance Test, IGT: Iowa Gambling Task, F = Female, SD: Standard-Deviation, Min: Minimum, Max: Maximum

For CPT variables, sex had no significant association with test performance (p>0.05). Age influenced the number of Commission Errors (r = -0.272, p<0.001) and Reaction Time (r = 0.410, p<0.001) but not Omission Errors (p=0.513). Education influenced Commission Errors (r=-0.181, p=0.012) and Response Time (r=0.180, p=0.012) but not Omission Errors (p=0.318). Age and Education were used as covariates on the Commission and Response Time Generalized Linear Models. Considering the IGT, no significant correlations were found between the Netscore and sociodemographic variables (p>0.05).

**Figure 1:**
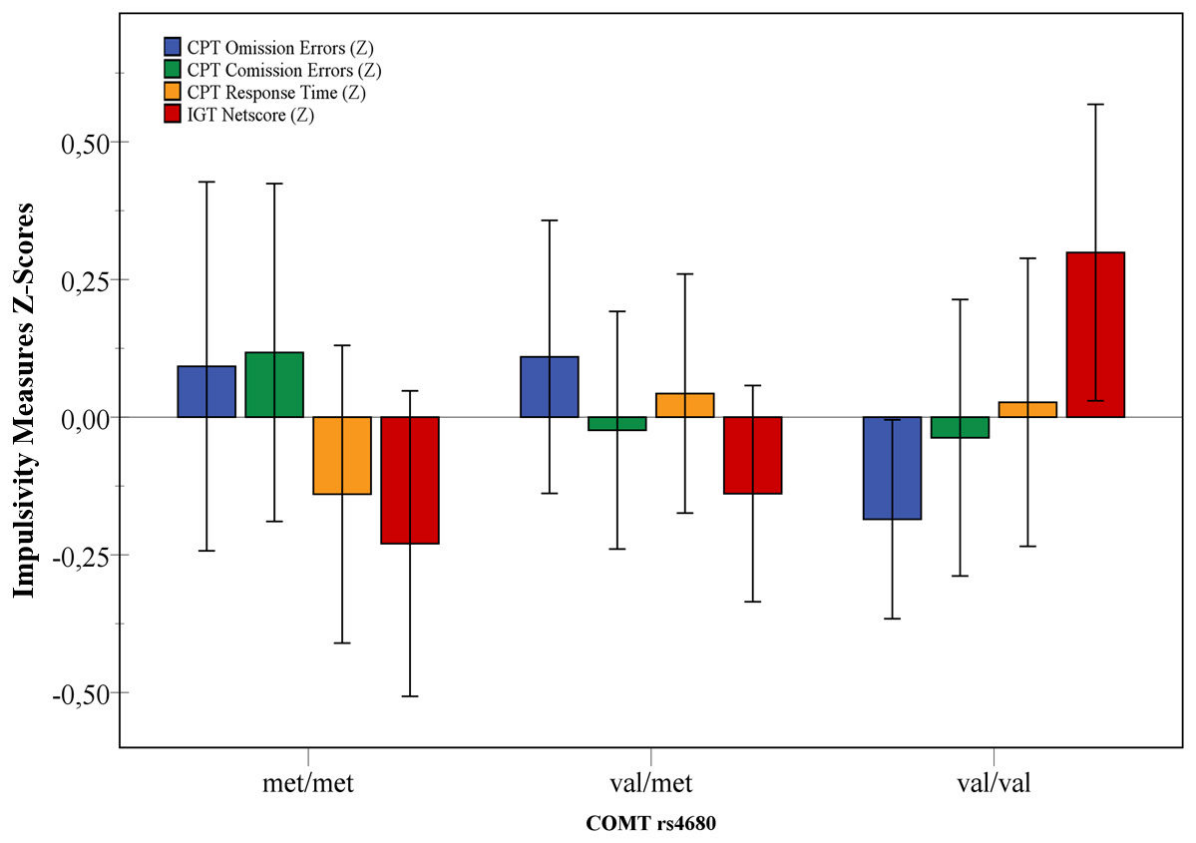
Mean Z-Scores for each Impulsivity measure along the COMT val158met groups. Significant differences were found on General Linear Models val homozygous when compared to val heterozygous (p=0.023) and met homozygous (p=0.019), with moderate effect sizes, suggesting a more impulsive response pattern by met carriers. No significant differences were found on CPT measures.

The Generalized Linear Model for CPT Commission errors was significant (χ^2^=13.76, df = 4, p=0.008), and age (χ^2^=5.62, df = 1, p=0.018) but not Education (χ^2^=2.83, df = 1, p=0.092) or COMT Genotype (χ^2^=0.80, df = 2, p=0.668) influenced test performance. The same pattern was observed for the CPT Response Time model (χ^2^=38.07, df = 5, p<0.001), with a significant influence of age (χ^2^=28.46, df = 1, p<0.001) but not Education (χ^2^=0.65, df = 1, p=0.420) and COMT Genotype (χ^2^=1.35, p=0.509) on motor impulsivity measures. The model of CPT Omission Errors was not significant (χ^2^=3.28, df = 2, p=0.152). Figure 1 shows the comparison of CPT impulsivity measures between the COMT Genotype Groups.

**Figure 2:**
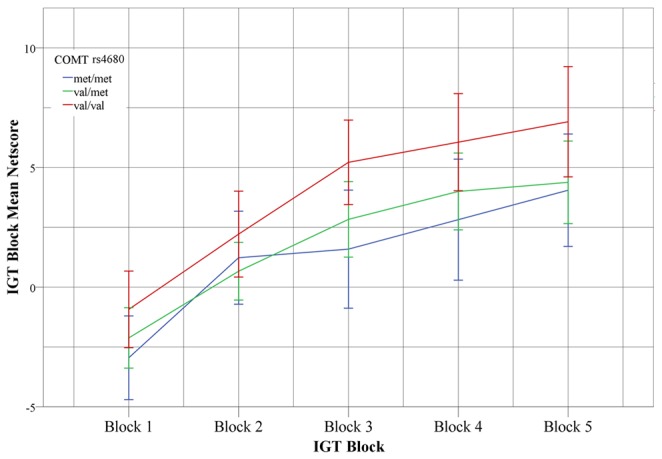
IGT Netscores along the five test blocks for COMT val158met groups. Along the IGT five blocks an learning curve occurs among the three genotypes, however, val homozygous shows a higher slope than met carriers, suggesting an less impulsive decision-making processes.

For the IGT Netscore, the General Linear Model containing only the COMT Genotype as a predictor was significant (F=5.13, p=0.007), with a moderate effect size (n^2^=0.05). Levene’s test suggests that the error variance was homogeneous across the COMT Genotype groups (F=1.65, p=0.194). Post-hoc analysis was performed using the conservative Sidak’s test, and Cohen’s d was adopted as an estimate of effect size. These results do not show significant differences between met/met and val/met groups (d=0.10, p=0.951), but significant differences were found when met/met and val/val (d=0.53, p=0.023) were compared, as well as for met/val and val/val (d=0.43, p=0.019), indicating that a more impulsive pattern of response was shown by met carriers. Effect sizes between val/val carriers and the other two COMT Genotype groups can be considered moderate. These comparisons are shown in Figure 1. The profile of IGT Netscore along the five test blocks is shown on Figure 2.

## Discussion

We investigated the relationship between the val158met COMT polymorphism and different aspects of impulsivity assessed by neuropsychological tests. Our results did not endorse the initial hypothesis of better performance in impulsivity measures for subjects homozygous for the met allele. The main finding in the present study was a relationship between the val/val homozygous allele and better performance in the decision-making task. At first glance, this result seems to be counterintuitive because the val allele is associated with higher COMT enzymatic activity and lower dopaminergic levels in the prefrontal cortex. Nonetheless, previous studies using the same neuropsychological task found similar results. Van Der Bos and colleagues [25] found that subjects with the met allele presented more disadvantageous choices in IGT. Other report [26] also described that the *rs4818* COMT G/G allele, which conferred higher COMT enzymatic activity, is related to a better performance on the IGT task. This finding is very similar to those reported here concerning the relationship between val allele and performance in IGT.

There are four possible explanations for this result. The first is related to the fact that an increase in dopaminergic levels in the prefrontal cortex in tonic stages of neurotransmission is accompanied by a reduction of dopaminergic levels in subcortical structures during phasic transmission. This situation leads to less flexible responses and impaired modulation in response to environmental novelty and emotional-derived processing [25]. Nonetheless, as demonstrated by other study [27], who did not find any association between IGT and the val158met polymorphism, IGT involves both cognitive and emotional components. Therefore, the lack of effect would not be expected. The dissociation between cognitive functions considered hot (more affective and emotional biased) and cool (more purely cognitive) and the val158met polymorphism should be addressed in future studies.

A second explanation for this result involves the relationship between dopamine and emotional/motivational valence to gains and reinforcement. Lancaster and colleagues [35] found that met/met allele carriers were more prone to reward-seeking behaviour than participants with val/val and val/met alleles in the balloon analogue risk task (BART). It is interesting to note that the authors also used a task to assess reward responsiveness. In this task, responses to one stimulus receive more frequent rewards than responses to another, leading to the development of a response bias characterised by an increasing chance to choose the more strongly reinforced stimulus. They found an association between reward responsiveness bias and BART scores in the responses of met/met subjects but not in the responses from val/met and val/val subjects [33].

Considering the Lancaster [33] results, we also found increased risk-taking behaviour in met/met allele subjects assessed in the present study. Although Lancaster did not report impairment in the cost-benefit analysis of the BART task, we found that met/met subjects improved their performance in the IGT over time. Lancaster reported that the cost-benefit analysis of met/met subjects was better than val/val and val/met subjects. Our results do not endorse this finding since met/met group scored worse on the IGT net score than the val/val and val/met groups. As noted by other study [36], BART and IGT are comparable but not equivalent tasks. In comparison to BART, IGT involves decision-making in an ambiguous context (i.e., the outcomes are not explicit) and gradual learning. To assess the hypothesis that the relationship between the met allele increases emotional/motivational valence, future studies should use a combination of risk and ambiguity decision-making tasks, such as IGT and BART. Furthermore, the use of concomitant IGT variations that measure sensitivity to reward and punishment as proposed previously [5] should be useful in elucidating the relationship between val158met and reward/punishment valence.

A third hypothesis is that the increasing dopaminergic brain levels does not necessarily account for better performance of prefrontal functions. In the present study, the performance of met/met subjects was the worst, which reinforces the hypothesis that an increased level of dopamine is not necessarily related to improvement in prefrontal functions [37,38]. It is also important to note that recent findings suggest that val158met polymorphism seems to play a role in the development of prefrontal white matter connectivity [38]. Data from studies using Diffusion Tensor Imaging (DTI) suggests that val allele is associated to a higher fractional anisotropy in prefrontal white matter tracts both in child, adolescents [39] and adults [40] than met allele. According to Thomason et al. [39], these structural white matter differences between val and met allele could be explained by the fact that increased dopaminergic levels could reduce human brain myelinisation processes. Future studies integrating neuroimage, genetics and neuropsychological data should be carried out to address the relationship between white matter connectivity, impulsivity and val158met polymorphism.

Finally, we can explain our hypothesis assuming a molecular double dissociation between the val and met alleles of the val158met polymorphism. According to a meta-analysis study [38], although the met allele is related to a better performance in tasks that assess processes involving purely executive function, the val allele is related to tasks influenced by emotional processing. Because IGT assesses affective decision-making, a process highly related to emotional/motivational function, our results reinforce this dissociation between the role of the met and val alleles in prefrontal cortex function. Mier and colleagues [38] argue that this dissociation is evidence of a pleiotropic behavioural effect of the COMT val158met polymorphism on executive function (where subjects homozygous for the val allele are less efficient) and executive control of emotion (where subjects homozygous for the met allele are less efficient).

We found that a specific component of impulsive behaviour related to decision-making presents a better performance in subjects homozygous for the val allele than in subjects homozygous for the met allele. However, we did not find differences between subjects grouped according to val and met alleles in CPT measures. Despite the strong association between the met allele and better performance in executive function tasks such as cognitive flexibility [41] and working memory [42], we did not find a relationship between impulsivity measures and COMT as predicted by a previous study [11]. This result is similar to Forbes and colleagues [18], who did not find an association between traits measured by BIS-11 and val158met, and Paloyellis and colleagues [19], who did not find an association between the BIS-11 total score in a sample of ADHD compared to normal adolescents. It is important to note, however, that even the absence of significant results between met and val carriers in CPT in the present study, due to the lack of previous studies that have used direct measures of motor and attentional impulsivity, we think that additional independent replication could contribute to elucidating the relationship between impulsivity and the val158met polymorphism.

This study includes some limitations. As in other study [35], we did not include intelligence assessment as inclusion criteria in the sample. Nonetheless, as we recruited subjects at a university we think that it is unlikely that the sample includes subjects with intellectual deficiency. Furthermore, intelligence seems to be unrelated to IGT performance [43]. Therefore, this variable does not appear to explain the genotype differences.

Another limitation of the study is that the relationship between dopamine and impulsive behaviour is much more complex than reported in the present study. Prefrontal processing is affected by both dopamine levels and receptor stimulation. Therefore, the impact and interaction of dopamine levels and receptors should be addressed by future studies of interactions between different genetic polymorphisms and dopaminergic function.

In addition, there is evidence that some aspects of impulsive behaviour are more related to striatal dopaminergic activity in comparison to prefrontal dopaminergic neurotransmission. For example, Forbes et al. [18], found that some dopaminergic polymorphisms (DRD2 -141C deletion; DAT1 9-repeat; DRD4 7-repeat), related to striatal dopaminergic neurotransmission, were associated to impulsivity self-reported measures and striatal reactivity paradigm. In the same study authors did not find any association between COMT val158met polymorphism and impulsivity measures. These data are, in some way, different from those reported in the present study. This difference could be explained by the fact that Iowa Gambling Task seems to be influenced by another executive functions processes more related to cortical dopaminergic activity as, for instance, working memory [44]. Future studies should address the dissociation between cortical *vs* subcortical dopaminergic neurotransmission and its relationship with different types of impulsive behaviour.

Concerning our sample two limitations must to be considered. First, the present sample is composed by students and is not a representative sample. Replication of the present study in a more representative sample and specific clinical groups is needed to assess the validity of the results. A second sample limitation is the imbalance of male/female ratio. The differences between impulsivity among males and females are controversial with both positive and negative findings [45–47]. In the present study impulsivity was not associated with gender and this data is in accordance with other studies that investigated the relationship between the val158met polymorphism and impulsivity in healthy adults [48]. Nonetheless, future studies, in a more representative sample should address the issue of a possible moderator/mediator role of gender in the relationship between val158met polymorphism and impulsive behaviour.

In conclusion, regardless of the limitations already mentioned, we consider that the present study made contributions to our understanding of the relationship between the COMT val158met polymorphism and a specific type of impulsive behaviour. On the other hand, we did not find a relationship between these polymorphisms and motor and attentional impulsivity. These data reinforce the hypothesis that different aspects of impulsivity are independent and stem from a different neurobiological basis. In comparison to previous studies that correlated IGT scores and the val158met polymorphism, we have recruited a larger sample. Future studies including patients affected by psychiatric disorders are required to clarify the relationship between impulsivity and the COMT val158met polymorphism.
